# Genomic epidemiology of methicillin-resistant and -susceptible *Staphylococcus aureus* from bloodstream infections

**DOI:** 10.1186/s12879-021-06293-3

**Published:** 2021-06-21

**Authors:** Joshua T. Smith, Elissa M. Eckhardt, Nicole B. Hansel, Tahmineh Rahmani Eliato, Isabella W. Martin, Cheryl P. Andam

**Affiliations:** 1 Department of Molecular, Cellular and Biomedical Sciences, University of New Hampshire, Durham, NH 03824 USA; 2 Dartmouth-Hitchcock Medical Center and Dartmouth College Geisel School of Medicine, Lebanon, NH 03756 USA; 3 Department of Chemical Engineering, University of New Hampshire, Durham, NH 03824 USA; 4 Department of Biological Sciences, University at Albany, State University of New York, Albany, New York, 12222 USA

**Keywords:** *Staphylococcus aureus*, Bloodstream infection, Genome evolution, Methicillin resistance, Multidrug resistance

## Abstract

**Background:**

Bloodstream infections due to *Staphylococcus aureus* cause significant patient morbidity and mortality worldwide. Of major concern is the emergence and spread of methicillin-resistant *S. aureus* (MRSA) in bloodstream infections, which are associated with therapeutic failure and increased mortality.

**Methods:**

We generated high quality draft genomes from 323 *S. aureus* blood culture isolates from patients diagnosed with bloodstream infection at the Dartmouth-Hitchcock Medical Center, New Hampshire, USA in 2010–2018.

**Results:**

In silico detection of antimicrobial resistance genes revealed that 133/323 isolates (41.18%) carry horizontally acquired genes conferring resistance to at least three antimicrobial classes, with resistance determinants for aminoglycosides, beta-lactams and macrolides being the most prevalent. The most common resistance genes were *blaZ* and *mecA*, which were found in 262/323 (81.11%) and 104/323 (32.20%) isolates, respectively. Majority of the MRSA (102/105 isolates or 97.14%) identified using in vitro screening were related to two clonal complexes (CC) 5 and 8. The two CCs emerged in the New Hampshire population at separate times. We estimated that the time to the most recent common ancestor of CC5 was 1973 (95% highest posterior density (HPD) intervals: 1966–1979) and 1946 for CC8 (95% HPD intervals: 1924–1959). The effective population size of CC8 increased until the late 1960s when it started to level off until late 2000s. The levelling off of CC8 in 1968 coincided with the acquisition of SCC*mec* Type IV in majority of the strains. The plateau in CC8 also coincided with the acceleration in the population growth of CC5 carrying SCC*mec* Type II in the early 1970s, which eventually leveled off in the early 1990s. Lastly, we found evidence for frequent recombination in the two clones during their recent clonal expansion, which has likely contributed to their success in the population.

**Conclusions:**

We conclude that the *S. aureus* population was shaped mainly by the clonal expansion, recombination and co-dominance of two major MRSA clones in the last five decades in New Hampshire, USA. These results have important implications on the development of effective and robust strategies for intervention, control and treatment of life-threatening bloodstream infections.

**Supplementary Information:**

The online version contains supplementary material available at 10.1186/s12879-021-06293-3.

## Background

Bacteremia refers to the presence of viable bacteria in the blood [1]. Asymptomatic bacteremia can occur from normal daily activities, such as vigorous toothbrushing or from minor medical and dental procedures [2]. Bacteremia is often transient and causes no further sequelae in healthy individuals [1]. However, when bacteria are able to breach innate host defenses and gain access to deeper tissues and subcutaneous sites, they can cause debilitating and life-threatening infections such as systemic inflammatory response syndrome, septic shock and organ failure [3]. Multiple bacterial species are implicated in bloodstream infections, of which *Staphylococcus aureus* cause 20.7% of total cases [4]. The incidence of bloodstream infections due to *S. aureus* is 20–50 per 100,000 population per year, with case-fatality rates of approximately 10–30% [5]. Bloodstream infection is notoriously hard to treat because it requires prompt source control and prolonged antimicrobial therapy [3]. Of major concern is the emergence and spread of multidrug-resistant and methicillin-resistant *S. aureus* (MRSA) in bloodstream infections, which are associated with therapeutic failure and increased mortality [6].

Understanding host and pathogen factors that impact bloodstream infections are crucial to disease control and treatment options. Host-related factors that determine outcome from bloodstream infections due to *S. aureus* have been widely studied and include age, comorbidities, immune status and primary focus of infection (i.e., the site of infection considered most likely to be responsible for seeding bacteria into the bloodstream) [3, 5]. The most prominent risk factor for bloodstream infections due to *S. aureus* is the presence of prosthetic devices and other kinds of medical implantation of foreign bodies that allow the bacterium to invade the intravascular space [3, 5]. However, the contribution of the bacterium in bloodstream infections is less understood. Previous studies report that *S. aureus* in the blood often derive from colonizing isolates from other parts of the body acquired prior to bloodstream infection such as the nasopharynx and gastrointestinal tract [7–9]. Different *S. aureus* clones in bloodstream infections may have adopted different strategies to overcome host responses such as cytolytic toxicity and biofilm formation [10]. An important gap in current knowledge is that relatively little is known of the genetic basis that underlies the successful adaptation and persistence of certain *S. aureus* lineages, in particular MRSA, in the larger population over the long term.

We generated high quality draft genomes from 323 *S. aureus* blood culture isolates from patients diagnosed with bloodstream infection at the Dartmouth-Hitchcock Medical Center, New Hampshire, USA in 2010–2018. We conclude that the *S. aureus* population was shaped mainly by the clonal expansion, recombination and co-dominance of two major MRSA clones in the last five decades.

## Methods

### Ethics approval

Ethical approval was granted by the Committee for the Protection of Human Subjects of Dartmouth-Hitchcock Medical Center and Dartmouth College. This study protocol was deemed not to be human subjects research. Samples used in the study were subcultured bacterial isolates that had been archived in the routine course of clinical laboratory operations. No patient specimens were used and patient protected health information was not collected.

### Bacterial isolates

Archived MRSA and methicillin-susceptible *S. aureus* (MSSA) isolates from 325 unique pediatric and adult patient blood stream infections were included in this study. These archived isolates grew from clinical blood culture specimens submitted to the Department of Pathology and Laboratory Medicine at the Dartmouth-Hitchcock Medical Center, New Hampshire, USA from December 2010 – August 2018. The first significant blood culture isolate from each patient is routinely archived (freezer space permitting) in case of future need for patient care, epidemiologic, public health or laboratory quality studies. Upon subculture, isolates were assigned a study number and all patient identifiers were removed with only the date of collection and results of clinical antimicrobial susceptibility testing linked to the study number. A convenience sampling composed of approximately half of the unique patient isolates distributed throughout the study period were randomly selected and included in our analysis. All isolates had previously been tested in vitro on an automated broth microdilution testing platform (MicroScan Walkaway, Beckman Coulter, Inc., La Brea, CA) against a panel of antimicrobial agents including cefoxitin (screening well), daptomycin, oxacillin, penicillin and vancomycin. Results were interpreted per Clinical Laboratory Standards Institute (CLSI) guidelines [11]. Methicillin resistance was determined per the manufacturer’s package insert and CLSI guidelines by growth in the cefoxitin screening well (> 4 μg/mL) and/or by an oxacillin minimum inhibitory concentration (MIC) of > 2 μg/mL. Methicillin-susceptible isolates were tested for beta-lactamase production using a nitrocefin disk (Remel, Lenexa, KS) using bacterial growth nearest to a 30 μg cefoxitin disk. Confirmatory penicillin zone-edge testing was not performed on beta-lactamase-negative isolates. Clinical breakpoints of other antimicrobials tested were interpreted per CLSI guidelines [11]. All isolates were stored in DMSO solution in − 80 °C.

### DNA extraction and whole genome sequencing

Isolates were subcultured from glycerol stocks onto commercially prepared tryptic soy agar with 10% sheep red blood cells (Remel, Lenexa, KS) and in brain heart infusion broth (BD Difco, Franklin Lakes, NJ) at 37 °C for 24 h. DNA was extracted and purified from the liquid culture using the Zymo Research QuickDNA Fungal/Bacterial Miniprep Kit (Irvine, CA) following manufacturer’s protocol. We used Qubit fluorometer (Invitrogen, Grand Island, NY) to measure DNA concentration. DNA libraries were prepared using the RipTide High Throughput Rapid DNA Library Prep kit (iGenomX, Carlsbad, CA). DNA samples were sequenced as multiplexed libraries on the Illumina HiSeq platform operated per the manufacturer’s instructions. Sequencing resulted to 250-nt long paired end reads. Sequencing was carried out at the UNH Hubbard Center for Genome Studies, Durham, NH, USA.

### De novo genome assembly, annotation and pan-genome construction

Reads were assembled into contigs using the Shovill v.1.1.0 pipeline (https://github.com/tseemann/shovill) with the option --trim to produce high quality draft genomes. Shovill is a series of methods that includes subsampling read depth down to 150X, trimming adapters, correcting sequencing errors and assembling using SPAdes [12]. We used the scaffolding and gap-filling programs SSPACE [13] and GapFiller [14] to improve the assemblies. Genome quality was assessed using the programs QUAST [15] and CheckM [16]. Overall, genomes were at least 97.7% complete with no more than 2.86% contamination. Only genomes with < 200 contigs and N50 of > 40 kb were included. The number of contigs range from 16 to 115 and N50 of 77–1011 kb (Additional file 1: Table S1 and Additional file 2: Fig. S1). After filtering out the genomes with low coverage and of poor quality, a total of 323 genomes were used for downstream analyses. We calculated the genome-wide average nucleotide identity (ANI) for all possible pairs of genomes using the program FastANI v.1.0 [17] and compared them to the reference strain (strain NCTC 8325; RefSeq assembly accession no. GCF_000013425.1). The resulting contigs were annotated using Prokka v.1.14.6 [18]. We used Roary v.3.13.0 [19] to characterize the totality of genes of all strains in a dataset or pan-genome [20].

### In silico sequence typing and detection of antimicrobial resistance

The sequence type (ST) of each isolate was confirmed using the program MLST v.2.19.0 (https://github.com/tseemann/mlst), which extracts seven housekeeping genes (*arcC, aroE, glpF, gmk, pta, tpi* and *yqiL*) from the sequence contigs and compares sequence variation against previously characterized STs in the *S. aureus* PubMLST database (https://pubmlst.org/organisms/staphylococcus-aureus/). We used ARIBA v.2.14.5 [21] and the ResFinder database [22] to identify resistance determinants due to horizontally acquired antimicrobial resistance genes. We also used VirulenceFinder [23] and the Virulence Factor Database (VFDB) [24] to screen all genomes for known virulence genes. We used SCCmecFinder v.1.2 [25] to identify the presence and type of *mecA*-carrying staphylococcal chromosomal cassette (SCC*mec*) using minimum thresholds of > 60% for sequence coverage and > 90% sequence identity.

### Phylogenetic and population structure analyses

Using the core genome alignment produced by Roary [19], we generated a maximum likelihood phylogeny using IQ-Tree v1.6.12 [26]. We used ModelFinder to select the most appropriate nucleotide substitution model [27]. We applied the general time reversible model of evolution [28] using nucleotide frequencies calculated directly from the sequence alignment and a FreeRate model of rate heterogeneity with three categories based on the ModelFinder results. We performed 1000 bootstrap replicates using ultrafast bootstrap approximation approach UFBoot2 [29]. We partitioned the strains on the core genome phylogeny into sequence clusters consisting of genetically similar individuals using the Bayesian hierarchical clustering algorithm RhierBAPS v1.1.2 [30].

### Construction of time-calibrated phylogenies

We identified single nucleotide polymorphisms (SNPs) in the full core genome length alignment using Snippy v.4.6.0 (https://github.com/tseemann/snippy) and mapped them to a reference genome (strain NCTC 8325). Nucleotide sites on the core SNP alignment of each sequence cluster that were identified by Gubbins v.2.4.1 [31] as having experienced recombination were removed to generate a phylogeny free of the distorting signal of recombination. To test for the presence of a temporal signal, we used the recombination-free phylogenies as input in the roottotip function in BactDating v1.0.12 [32] to perform a root-to-tip linear regression analysis and random permutation of sampling dates to assess significance. We used BactDating to estimate the dates of the most recent common ancestor. We carried out 10^8^ iterations, removed the first half as burnin and the remainder was sampled every 100 iterations. Markov Chain Monte Carlo (MCMC) chain lengths were carried out until effective sample sizes of the inferred parameters α (coalescent time unit), μ (mean substitution rate) and σ (standard deviation of the per-branch substitution rates) parameters reached > 200 as suggested by the authors. To estimate the effective population size (Ne) for the time-scaled phylogenies, we used the R package [33] Skygrowth v. 0.3.1 that implements a Bayesian MCMC and maximum a posteriori algorithms [34]. Skygrowth models the growth rate of Ne as a simple Brownian motion process.

### Estimating recombination rates

Using the core genome alignment as input and 1000 bootstrapped replicates, we calculated the recombination rate using mcorr with default parameters [35]. Mcorr uses a coalescent-based model to measure the degree to which any two loci separated by N bp have correlated substitutions and estimates six evolutionary parameters: θ – mutational divergence; ϕ – recombinational divergence; c – recombination coverage or proportion of sites in the genome whose diversity was derived from outside the sample through recombination; d – diversity; mean fragment size (f̅) of a recombination event; and θ/ϕ (or γ/μ) - relative rate of recombination to mutation [35]. We used Welch’s t-test to compare the mcorr parameters between samples.

Unless otherwise noted, default parameters were used for all programs.

## Results

### Phylogenetic relationships and population structure

**Fig. 1:**
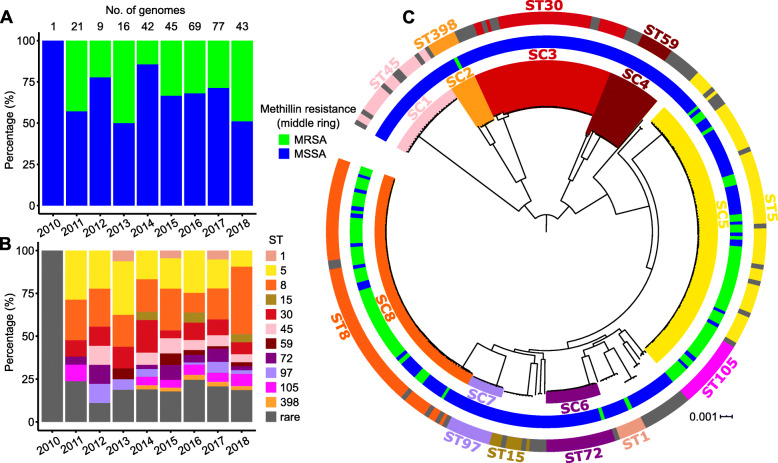
Methicillin resistance and phylogenetic relationships of *S. aureus* isolates. **a** Yearly sampling of methicillin resistance based on either cefoxitin or oxacillin phenotypic screenings. **b** Yearly distribution of sequence types (ST) throughout the study period. Gray bars indicate STs either previously unknown or rare (< 3 individuals). **c** Midpoint-rooted maximum likelihood tree showing phylogenetic structure of 323 *S. aureus* isolates. Clade colors on the branches represent sequence clusters (SC) defined by the BAPS program. Inner ring shows the phenotypic results of methicillin resistance screening as described above. Outer ring shows the distribution of different STs

We obtained a total of 325 convenience MRSA and MSSA isolates recovered from unique pediatric and adult patient blood cultures at the Dartmouth-Hitchcock Medical Center, USA which were sampled from December 2010 to August 2018. In vitro phenotypic susceptibility testing revealed a total of 105 isolates (32.31%) that were MRSA. We observed the occurrence of MRSA every year, with prevalence ranging from 14.3% in 2014 to 48.8% in 2018 (Fig. 1a). We retrieved high-quality draft genome sequences from 323 isolates (Additional file 1: Table S1 and Additional file 2: Fig. S1). De novo genome assembly produced sequences ranging from 2.67 to 2.97 Mb in length. Annotation of assembled genomes revealed a total of 8430 families of orthologous genes, which can be classified into 1698 core genes (genes present in 320–323 genomes), 171 soft core genes (present in 307–319 genomes), 1369 shell genes (present in 49–306 genomes) and 5192 cloud genes (present in ≤48 genomes) (Additional file 3: Table S2 and Additional file 4: Fig. S2). The number of genes per genome spanned from 2440 to 2814. The sizes of the pan- and core genomes were consistent with findings from previous genomic studies of *S. aureus* in clinical and non-clinical settings [36, 37].

Multilocus sequence typing (MLST) revealed high *S. aureus* diversity throughout the duration of our study period, with at least five sequence types (STs) present every year and several rare and previously unidentified STs (Fig. 1b and Additional file 1: Table S1). Those STs that were consistently observed from 2011 to 2018 were STs 5, 8 and 30, while STs 1 and 15 were observed in alternating years. ST398 appeared first in 2014 and continued until 2018. Other STs such as STs 45, 59, 72, 97 and 105 were less common and appeared intermittently over 8 years of our sampling period.

Population structure analysis using Bayesian hierarchical clustering of the core genome alignment showed eight distinct sequence clusters, ranging in size from 8 to 89 isolates (Fig. 1c). Sequence clusters consist of genetically related strains of the same or closely related STs. The population was dominated by two large clusters. Sequence cluster 5 consisted mainly of STs 5 and 105 (which are members of clonal complex (CC) 5 [38]), while sequence cluster 8 consisted mostly of ST 8 (which is a member of CC8 [39]). The majority of the MRSA identified using in vitro phenotypic testing were present in these two clusters [50/89 (56.18%) in CC5 and 52/76 (68.42%) in CC8]. We also identified one MRSA isolate in each of sequence clusters 2 (ST398), 6 (ST72) and an undelineated cluster consisting of ST1. Isolates that did not group with the eight sequence clusters made up the low frequency genotypes that were divergent from the rest of the population and with each other.

### Resistance to multiple antimicrobial classes

**Fig. 2:**
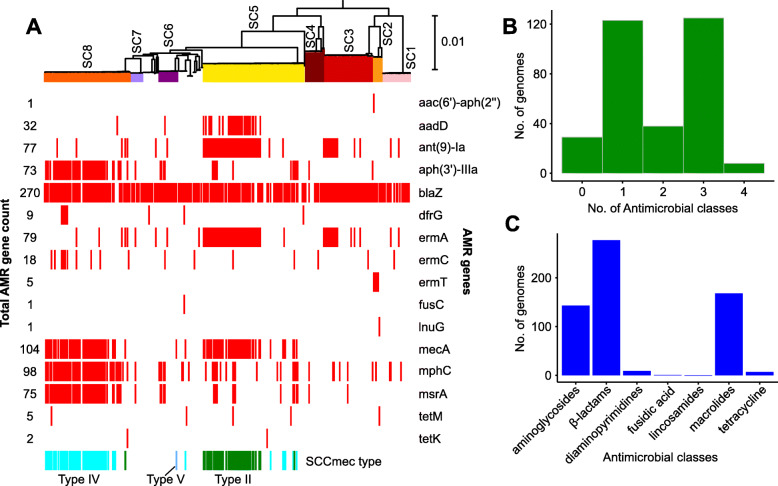
Antimicrobial resistance gene and class distribution among 323 isolates. **a** Gene presence-absence matrix showing the distribution of antimicrobial resistance genes and SCC*mec* types across the phylogeny (tree is identical to that in Fig. 1c). Red blocks indicate presence of gene listed to the right of the panel. SCC*mec* types are color-coded as green = Type II, aqua = Type IV, blue = Type V. **b** Histogram showing distribution of the number of antimicrobial classes each genome is resistant to. **c** Bar chart showing the number of genomes resistant to each individual antimicrobial class.

We considered in silico evidence to identify resistance determinants in each isolate (Fig. 2a and Additional file 5: Table S3). We detected a total of 20 horizontally transferrable resistance genes. Two mechanisms mediate penicillin resistance in *S. aureus*. The *blaZ* gene encodes a narrow-spectrum penicillinase, which inactivates the penicillinase-labile penicillins (penicillin, amoxicillin, ampicillin, carbenicillin, ticarcillin, azlocillin and piperacillin) by hydrolysis of its β-lactam ring [40]. The *mecA* gene encodes an alternative penicillin-binding protein PBP2a and confers resistance to all beta-lactam antimicrobial agents with the exception of ceftaroline [41]. We detected *blaZ* and *mecA* in 262/323 isolates (81.11%) and 104/323 isolates (32.20%), respectively. We found a slight discrepancy between the in silico detection of the *mecA* gene and in vitro phenotypic testing for methicillin resistance. There were two isolates whose genomes contained the *mecA* gene but phenotypically tested as methicillin-susceptible. There were three isolates whose genomes did not contain the *mecA* gene but phenotypically tested as MRSA. These phenotype/genotype discrepancies may be due to sequencing errors, the existence of novel *mecA* homologs that are not recognized as yet in current databases or the presence of other yet unknown determinants of methicillin resistance. The *mecA* gene is carried by the mobile element staphylococcal cassette chromosome (SCC*mec*), of which there are currently 14 recognized types [42]. In our study, SCC*mec* type II is associated mainly with CC5 (43/89 or 48.31% of total isolates in the cluster), while type IV with CC8 (52/76 or 68.42% of total isolates in the cluster). Each of the two CCs also contains isolates with SCC*mec* type from the other CC (i.e., five isolates in CC5 have SCC*mec* type IV and one isolate in CC8 have SCC*mec* type II). We also detected SCC*mec* type V in sequence cluster 6 (ST72). The three SCC*mec* types vary in terms of their gene content [43]. SCC*mec* type II is a relatively large element (~ 52 kb) carrying a type 2 recombinase-encoding *ccr* gene complex and is commonly found among healthcare-acquired MRSA strains, including CC5 [43, 44]. It carries transposon Tn554, which contains the antimicrobial resistance genes *ermA* (erythromycin resistance) and *spc* (spectinomycin resistance). SCC*mec* Type IV is the smallest type (~ 24 kb), often circulating in community settings [43]. Often, this element contains transposon Tn4001, carrying genes conferring resistance to aminoglycosides [43]. Like type IV, SCC*mec* type V is smaller in length (~ 27 kb) and is often found in community-associated MRSA [43]. It carries other resistance genes aside from *mecA*, but has a restriction modification system that may play a role in stabilizing the SCC*mec* [43].

We identified other horizontally acquired resistance genes [45] from the genome sequences. These were differentially distributed across the population and in some cases were associated mainly with specific sequence clusters. For example, the genes *aadD* (kanamycin resistance), *ant(9)-Ia* (aminoglycoside resistance) and *ermA* (erythromycin resistance) were mainly found in CC5 [30/89, 53/89 and 52/89 genomes, respectively]. The genes *aph(3′)-IIIa* (aminoglycoside resistance), *mphC* (macrolide resistance) and *msrA* (macrolide and streptogramin B resistance) were most frequently observed in isolates from CC8 (54/76, 56/76 and 55/76 genomes, respectively) [45]. Although the prevalence of vancomycin resistance had been reported to be increasing in *S. aureus *in recent years [46], we did not detect vancomycin-resistant isolates using either phenotypic assays or in silico methods. Overall, we found that 133/323 isolates or 41.18% carry horizontally acquired genes conferring resistance to at least three antimicrobial classes (Fig. 2b), with resistance determinants for aminoglycosides (143/323), beta-lactams (277/323) and macrolides (168/323) being the most prevalent (Fig. 2c).

### Virulence characteristics of *S. aureus* genomes

We used in silico screening of virulence genes that may play a critical role in bloodstream infections (Additional file 6: Fig. S3 and Additional file 7: Table S4). Several of these were detected among different sequence clusters. The *hlgABC* pore-forming leucotoxin genes were found in every genome, with the exception of two genomes missing the *hlgC* gene. We found the *sak* gene, which encodes a plasminogen activator protein staphylokinase [47], in 297/323 genomes (or 91.95%). The gene product of *sak* has two known roles in virulence. First, it forms a complex with plasmin to prevent biofilm formation in the blood, allowing for access to deeper tissues [47]. It also prevents antimicrobial peptides excreted by the host to effectively target bacterial cells [47]. Found in 309/323 genomes (or 95.67%), the virulence factor staphylococcal complement inhibitor encoded by the *scn* gene allows *S. aureus* to evade phagocytosis and killing by human neutrophils [48]. The metalloproteinase-encoding aureolysin gene (*aur*) was found in 322/323 genomes (99.69%). Aureolysin has been implicated in the inactivation of host protease inhibitors and degradation of host antimicrobial peptides [49]. Two allelic variants of *aur* have been previously reported [50] and we found both variants in our dataset: *aur* in 246/323 genomes (76.16%) and *aur1* in 76/323 genomes (23.53%). We detected the *aur* variant in sequence clusters 4, 5, 6, 7, 8 and the unclustered strains, while *aur1* was found in sequence clusters 1, 2 and 3. There appears to be little functional difference between the two *aur* variants and it remains unclear why they continue to persist in distinct *S. aureus* lineages [50].

We found differences in the distribution of virulence genes between CC5 and CC8. For example, CC5 has a much larger number of enterotoxin genes compared to CC8. Enterotoxins are proteins secreted by primarily by *Staphylococcus* spp. that target intestinal tissues and are commonly associated with food poisoning and toxic shock syndrome [51]. Among these enterotoxin-encoding genes, we detected *seg, sei, sem, sen, seo* and *seu* in every CC5 genome with very few found in CC8 genomes. Other genes such as *sed, sej* and *ser* were also found more frequently in CC5 (46/89, 49/89 and 48/89 genomes, respectively). In contrast, the enterotoxin genes *sek* and *seq* were more common in CC8 (48/76 genomes for both). The gene *splE*, which encodes a serine protease [52], was also common in CC8 (71/76 genomes). The Panton-Valentine leukocidin (PVL) genes *lukF* and *lukS* were frequently associated with CC8 (48/76 and 47/76 genomes, respectively). The prophage-encoded PVL produces a pore-forming toxin capable of killing neutrophils, monocytes and macrophages and is closely linked to severe *S. aureus* infections [53]. We also detected the arginine catabolic mobile element genomic island that can enhance the growth and survival of *S. aureus* [54] in 47/76 genomes in CC8.

Among the other sequence clusters, one notable finding is the presence of the gene that encodes the superantigen toxic-shock syndrome toxin-1 (TSST-1) among 39/43 or 90.70% of the genomes in ST30. TSST-1 activates T-cells, which results to the induction of a cytokine storm and can become fatal [55].

### Estimating the date of clonal origins and effective population size

**Fig. 3:**
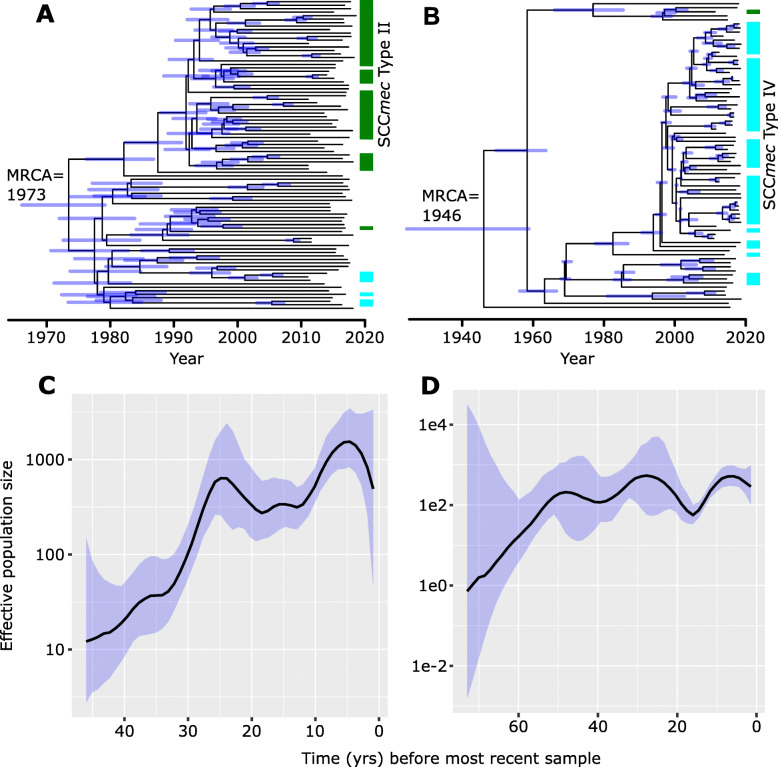
Bayesian phylogeny and population dynamics of CC5 and CC8. Bayesian maximum clade credibility time-calibrated phylogeny of CC5 (**a**) and CC8 (**b**) based on non-recombining regions of the core genome. Divergence date (median estimate with 95% highest posterior density dates in brackets) is indicated in blue on the tree. Bayesian skygrowth plot showing changes in effective population size (Ne) over time of CC5 (**c**) and CC8 (**d**). Median is represented by a black line and 95% confidence intervals are in blue

We sought to determine the dates of clonal origin of CCs 5 and 8, which make up majority of the MRSA genomes. Using the recombination-free alignment of core SNPs as input to BactDating [32], we first determined the presence of a temporal signal in each CC (Additional file 8: Fig. S4). We observed a significant positive correlation between the dates of isolation and root-to-tip distances (CC5: *R*^*2*^ = 0.06 and *p* value = 1.4 e-2 and CC8: *R*^*2*^ = 0.15 and *p* value = 3.0 e-4) indicating the presence of a clock-like signal. Results showed that the two CCs emerged in the New Hampshire population at separate times. We estimated that the time to the most recent common ancestor of CC5 was around 1973 (95% highest posterior density (HPD) intervals: 1966–1979) and around 1946 for CC8 (95% HPD intervals: 1924–1959) (Fig. 3ab). For each CC, we also estimated the change in the effective population size (Ne), which is a measure of the rate of change in population composition due to genetic drift [56]. The effective population size of CC8 increased until the late 1960s when it started to level off until late 2000s. The onset of the levelling off of CC8 in 1968 coincided with the acquisition of SCC*mec* Type IV in majority of the strains. The plateau in CC8 also coincided with the acceleration in the population growth of CC5 in the early 1970s and which eventually leveled off in the early 1990s. The beginning of the levelling off of CC5 in 1993 overlaps with the acquisition of SCC*mec* Type II (Fig. 3 cd). These results indicate that the emergence of lineages each carrying distinct SCC*mec* types contributed to the success of the two MRSA CCs in the local population.

### Variation in recombination rates

**Fig. 4:**
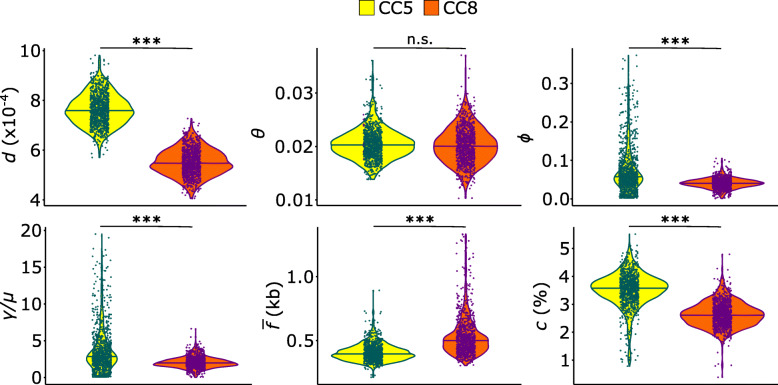
Recombination parameters comparing CC5 and CC8. Core genome alignment of each CC was used as input in mcorr with 1000 bootstrapped replicates. d – diversity brought into the population by recombination and clonal diversity; θ – mutational divergence; ϕ – recombinational divergence; γ/μ relative rate of recombination to mutation (equivalent to ratio of ϕ/θ); f̅ – mean fragment size of a recombination event; c – recombination coverage. For all panels, brackets indicate results of Welch’s t-test of group comparisons; n.s. - not significant; *** - *p* < 0.0001

Homologous recombination, whereby similar tracts of DNA are acquired from closely related strains [57, 58], is known to impact the evolution and adaptation of *S. aureus* to its hosts [59–61]. The genetic diversity generated through recombination may be further disseminated in the population via subsequent recombination events with other strains or may be passed on to descendants through clonal descent. Here, we sought to determine the extent in which recombination has affected the genome evolution of the two dominant MRSA clones CCs 5 and 8. Using the core genome alignment as input and 1000 bootstrapped replicates, we used mcorr to calculate six recombination parameters (Fig. 4 and Additional file 9: Table S5) [35]. The diversity (d) is the probability that a pair of genomes will differ at any locus and is estimated from the diversity generated from both recombination and accumulation of mutations of the clonal lineage. This parameter was estimated to be 7.63 × 10^− 4^ and 5.51 × 10^− 4^ in CC5 and CC8, respectively (*p* value = 0.0; Welch’s t test). The mutational divergence (θ), which refers to the mean number of mutations per locus since divergence of a pair of homologous sites, was estimated to be 0.020 for both CC5 and CC8 (*p* value = 0.120; Welch’s t test). Recombinational divergence (ϕ) was estimated to be 0.073 and 0.041 in CC5 and CC8, respectively (*p* value = 4.19 × 10^− 58^; Welch’s t test). The ratio ϕ/θ (or γ/μ), which gives the relative rate of recombination to mutation, was estimated to be 3.63 and 2.06 in CC5 and CC8, respectively (*p* value = 1.28 × 10^− 53^; Welch’s t test). The mean fragment size (f̅) of a recombination event was estimated to be 407.45 bp and 530.55 bp in CC5 and CC8, respectively (*p* value = 4.30 × 10^− 107^; Welch’s t test). Lastly, we calculated the recombination coverage c, which indicates the fraction of the genome whose diversity was derived from recombination events since the last common ancestor of the population, and which ranges from 0 (indicating clonal evolution) to 1 (indicating complete recombination) [35]. Recombination coverage was estimated to be 0.035 and 0.026 in CC5 and CC8, respectively (*p* value = 1.10 × 10^− 231^; Welch’s t test), indicating that 3.5 and 2.6% of sites in any one genome from CC5 and CC8, respectively, originated from recombination events. Except for the mutational divergence (θ), we found significant differences in five recombination parameters between the two clonal complexes. We compared these values to those calculated by the authors of mcorr for a *S. aureus* genomic dataset of 308 invasive isolates from 26 countries in Europe and representing 10 CCs and six minor STs [62]. Both CC5 and CC8 in the New Hampshire population have lower diversity (0.015 in Europe), mutational divergence (0.042 in Europe) and coverage (0.36 in Europe) than the European *S. aureus*, but higher γ/μ (1.0 in Europe). These were expected because the European dataset consisted of more diverse genotypes. Remarkably, the recombinational divergence and mean recombination size of the European *S. aureus* were 0.042 and 550, respectively, which were similar to the values of the New Hampshire CC8. CC5 had higher recombinational divergence and lower mean recombination size than the European *S. aureus*. Overall, these results indicate that the two MRSA clones in New Hampshire have experienced frequent recombination during their recent clonal expansion.

## Discussion

Elucidating the evolutionary context that has selected certain methicillin-resistant *S. aureus* genotypes is critical to developing effective surveillance, control strategies and treatment options for bloodstream infections. Using genome sequences of 323 *S. aureus* blood culture isolates spanning at least eight years of sampling, our analyses revealed the specific bacterial clones that predominate in bloodstream infections (CCs 5 and 8) and the genomic elements that contribute to their success as multidrug resistant pathogens in bloodstream infections.

Two mechanisms explain the spread of drug resistance in bacterial populations: clonal expansion of resistant lineages [63–66] and horizontal gene transfer of resistance genes [67–69]. Our study showed that both processes have shaped the long-term population dynamics of MRSA in New Hampshire. Our first major finding is that both CC5 and CC8 (each carrying different suites of resistance genes) have undergone clonal expansion in the last 50 years, which would partly explain the broad occurrence of multidrug and methicillin resistance in the *S. aureus* population. The clones that comprise these two lineages are the most prominent MRSA lineages worldwide and have been implicated in numerous healthcare-associated and community-acquired infections and outbreaks [44]. CC8, which includes ST8, consists of several pandemic MRSA clones associated with healthcare- and community-associated infections [39, 44]. ST8 includes the notorious pandemic clone USA300 that emerged in Central Europe in the mid-nineteenth century and was exported to North America and elsewhere in the early twentieth century [70]. Community and household transmission facilitated the widespread distribution of this clone in the United States [71–73]. ST8 also includes USA500 that emerged in the past 20 years and causes invasive infections in North America [74]. On the other hand, CC5 includes multiple MRSA clones that cause hospital-associated infections in the Western Hemisphere [38]. It is also the principal genetic background in *S. aureus* upon which full resistance to vancomycin, a key last-line bactericidal drug for treating MRSA infections, has arisen by acquisition of the Tn1546 carrying the *van* operon from *Enterococcus* donors [75]. Although none of the New Hampshire isolates were vancomycin-resistant, reduced vancomycin susceptibility has been increasing in *S. aureus* causing bloodstream infections in other parts of the United States [46, 76] and it may therefore just be a matter of time before it emerges in New Hampshire.

The predominance of CCs 5 and 8 in bloodstream infections has been previously reported in other places. In Minnesota USA, the most common MRSA clones were CC5 (ST5, all SCC*mec* type II) and CC8 (ST8 and ST3342, mostly SCC*mec* type IV) [77]. In a Colorado USA study that tracked *S. aureus* colonizing the nares and progressing to bacteremia within individual patients, STs 5 and 8 were also the most frequently observed [9]. In a study of MRSA isolates causing bloodstream infections in England sampled from 2012 to 2013, ST22 (clonal complex 22) make up the majority of *S. aureus* isolates, while STs 5 and 8 were less common [78]. In nine Latin American countries sampled in 2011–2014, the population of *S. aureus* from bloodstream infections was dominated by STs 5 and 8 as well as minor STs such as STs 30 and 72 [79]. In contrast to our results, the Latin American study reported evidence for frequent clonal replacement of previously prevalent hospital-associated clones: ST8 replaced ST5 in Colombia and Ecuador, while ST5 replaced ST239 in Brazil [79]. In our New Hampshire study, co-dominance rather than clonal replacement enabled both methicillin resistant CC5 (ST5) and CC8 (ST8) to persist in the last five decades. We can only postulate the reasons for such remarkable contrast in *S. aureus* population dynamics – clonal replacement in South America versus clonal co-dominance in North America – in a relatively short time span. Selective pressures due to ecological differences may explain this variation. Differences in the population dynamics of resistant bacteria could be due to regional differences in host immunity that selected for specific genes that happened to be resistant, differences in antimicrobial use or different patterns of transmission among specific host groups that receive more antimicrobial prescriptions such as children [80]. Another important consideration is that our dataset included both pediatric and adult patients, while the Latin American study consisted only of adult patients. The size and diversity of the geographical regions being compared (New Hampshire as a single state in the United States versus multiple countries in Latin America) will also greatly influence these results. Lastly, while the sampling period (2010–2018 in our study versus 2011–2014 in the Latin American study) overlapped, making definitive comparison between the two remains challenging because of disparities in sampling strategies. Regardless, the co-dominance of two high-risk clones with distinct resistance and virulence characteristics is an important consideration in the epidemiology of *S. aureus* causing bloodstream infections in New Hampshire.

Our second major finding is that the second mechanism, i.e., horizontal acquisition of resistance genes, was also a major driver in the spread of resistance in *S. aureus* causing bloodstream infections. Although the mobile *mecA* was primarily found in CC5 and CC8, numerous horizontally-acquired resistance genes were also differentially distributed in other lineages. Two possible scenarios can happen. These rare lineages (i.e., isolates that were not part of CCs 5 and 8) can act as reservoir of resistance genes and their allelic variants from which the dominant lineages can draw from [44, 81, 82]. It may also be possible that the presence of these resistance genes can facilitate the clonal expansion and dissemination of the rare genotypes in the future [83, 84]. In either case, these rare genotypes carrying horizontally mobile resistance genes must be observed over the long term to ensure that risks due to either scenario are reduced. Moreover, both CC5 and CC8 appear to have had a history of frequent recombination during their recent clonal expansion. Frequent or large-scale genetic recombination events can significantly alter the genome structure of a strain, which can drive the emergence of lineages with unique traits such as ability to switch between animal and human hosts, epidemic features or enhanced growth in new ecological niches [61, 82, 85, 86].

We acknowledge several limitations of our study. First, only one isolate was obtained per patient. Multiple infections (either repeated or mixed infections) and the diversity of the *S. aureus* inoculum at the site of infection can greatly influence the *S. aureus* population within a single patient as the pathogen evolves during the course of infection [87]. Second, characterization of mobile elements, resistance genes and virulence genes depend greatly on the composition and quality of existing databases used in in silico methods. This means that novel genetic variants (including unknown types of SCC*mec*) may not be recognized by current methods. Lastly, as in any phylogenetic and population demographic method, estimation of the dates of the last common ancestor is made difficult with increasing time between the ancestor and the observed descendants as well as the range of diversity of missing descendants. Hence, the dates of the last common ancestors of CC5 and CC8 represent only an approximation and may vary with the inclusion of additional isolates.

The results presented here open multiple avenues for future research. Continuous surveillance over many years is essential for documenting the long-term dynamics and drug resistance of *S. aureus*, including the potential for clonal replacement by other, less common lineages. Low frequency clones, which may harbor unique genomic elements or phenotypic features, may remain hidden from epidemiological and phylogenetic studies. They also have the capacity to replace one or both co-dominant lineages (CCs 5 and 8), which may be facilitated by changes in ecological conditions (e.g., change in antimicrobial use and demographic changes in the region). Future surveillance strategies will also be enhanced by the inclusion of other hospitals in the region. It is likely that a more heterogeneous assembly of *S. aureus* lineages are present in New Hampshire, but the lack of a systematic state- or region-wide *S. aureus* surveillance in bloodstream infections may lead to a subpopulation of genetic variants that we may have overlooked in the present analyses.

Second, future work should also focus on the relationship of clone-specific genomic features and the clinical outcomes of patients with bloodstream infection, which can be used as predictive factors in *S. aureus* mortality, clinical manifestation and disease severity. Once *S. aureus* enters the bloodstream, it replicates and disseminates to many different sites, causing severe disease manifestations such as sepsis, infective endocarditis, and deep-seated abscesses in virtually every organ tissue [3]. It remains unclear if there is site-specific selection occurring for specific strains or genetic variants. This information will be particularly useful to precisely characterize protective vaccine antigens and the development of immune therapeutics to prevent disease or improve clinical outcomes. It will also inform appropriate patient management and therapeutics for persistent, relapse, metastatic or complicated *S. aureus* bloodstream infections. Unfortunately, our data set does not include an extensive amount of clinical, phenotypic or other epidemiological information for each isolate. Hence, we strongly advocate for the inclusion of such pertinent associated data in sampling and surveillance schemes of bloodstream infections in the state of New Hampshire and elsewhere.

## Conclusions

We conclude that the *S. aureus* population causing bloodstream infections in New Hampshire, USA was shaped mainly by the clonal expansion, recombination and long-term co-dominance of two high-risk lineages with distinct genomic features, resistance characteristics and evolutionary histories. These results have important implications on the development of effective and robust approaches to identifying new bacterial targets for intervention, control and treatment strategies of life-threatening bloodstream infections.

## Supplementary Information

**Additional file 1: Table S1.** Genome assembly, MLST, accession numbers and associated metadata of 323 *S. aureus* isolates

**Additional file 2: Fig. S1.** Assembly statistics of the *S. aureus* genomes

**Additional file 3: Table S2.** Distribution of genes in the *S. aureus* pan-genome identified using Roary

**Additional file 4: Fig. S2.** Pan-genome analysis of 323 *S. aureus* genomes. (a) Presence-absence matrix of gene clusters as determined by Roary, aligned to the phylogeny. Blue indicates presence. (b) Gene frequency histogram indicating the number of genomes each gene is present in. (c) The size of the pan-genome (blue), core genome (red), unique gene additions (purple) and new genes (green) as related to the number of individuals in the population. Proportional illustration of core, soft-core, shell, and cloud genes across the pangenome.

**Additional file 5: Table S3.** Distribution of horizontally acquired antimicrobial resistance genes identified using ResFinder

**Additional file 6: Fig. S3.** Distribution of virulence genes using VirulenceFinder and the Virulence Factor Database (VFDB). Details are shown in Supplementary Table S4.

**Additional file 7: Table S4.** Distribution of virulence genes identified using VirulenceFinder and the VFDB

**Additional file 8: Fig. S4.** Bactdating statistical tests and MCMC trace plots. (a) Initial rooted phylogeny and correlation test between date and root-to-tip distance withing the phylogeny for CC5. (b) Bactdating trace plots constructed by periodic sampling over the MCMC runs for CC5. (c) Initial phylogeny and correlation test for CC8. (d) Bactdating trace plots for CC8.

**Additional file 9: Table S5.** Recombination parameters inferred by mcorr for each of CC5 and CC8

## Data Availability

The dataset supporting the conclusions of this article is included within the article and its additional files. Genome sequence data of the *S. aureus* blood culture isolates have been deposited in the National Center for Biotechnology Information (NCBI) Sequence Read Archive under BioProject accession number PRJNA673382 with BioSample accession numbers for each genome listed in Additional file 1: Table S1. The reference strain NCTC 8325 (RefSeq assembly accession no. GCF_000013425.1) is available in NCBI.
