# Supplementary figures and images for: Genomic epidemiology of methicillin-resistant and -susceptible Staphylococcus aureus from bloodstream infections
Source: BMC Infect Dis. 2021 Jun 21;21:589. doi: 10.1186/s12879-021-06293-3 (PMC8215799; doi:10.1186/s12879-021-06293-3)

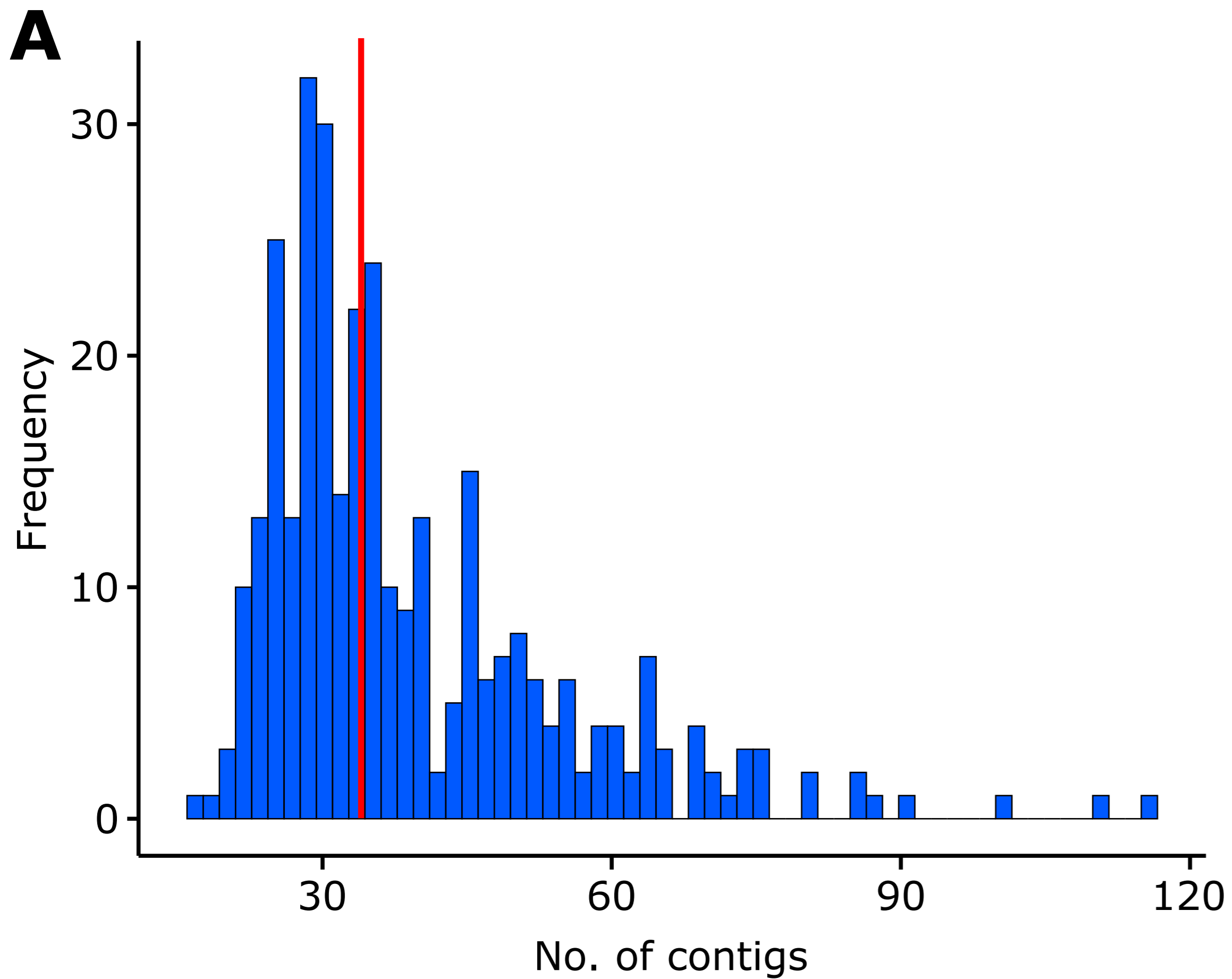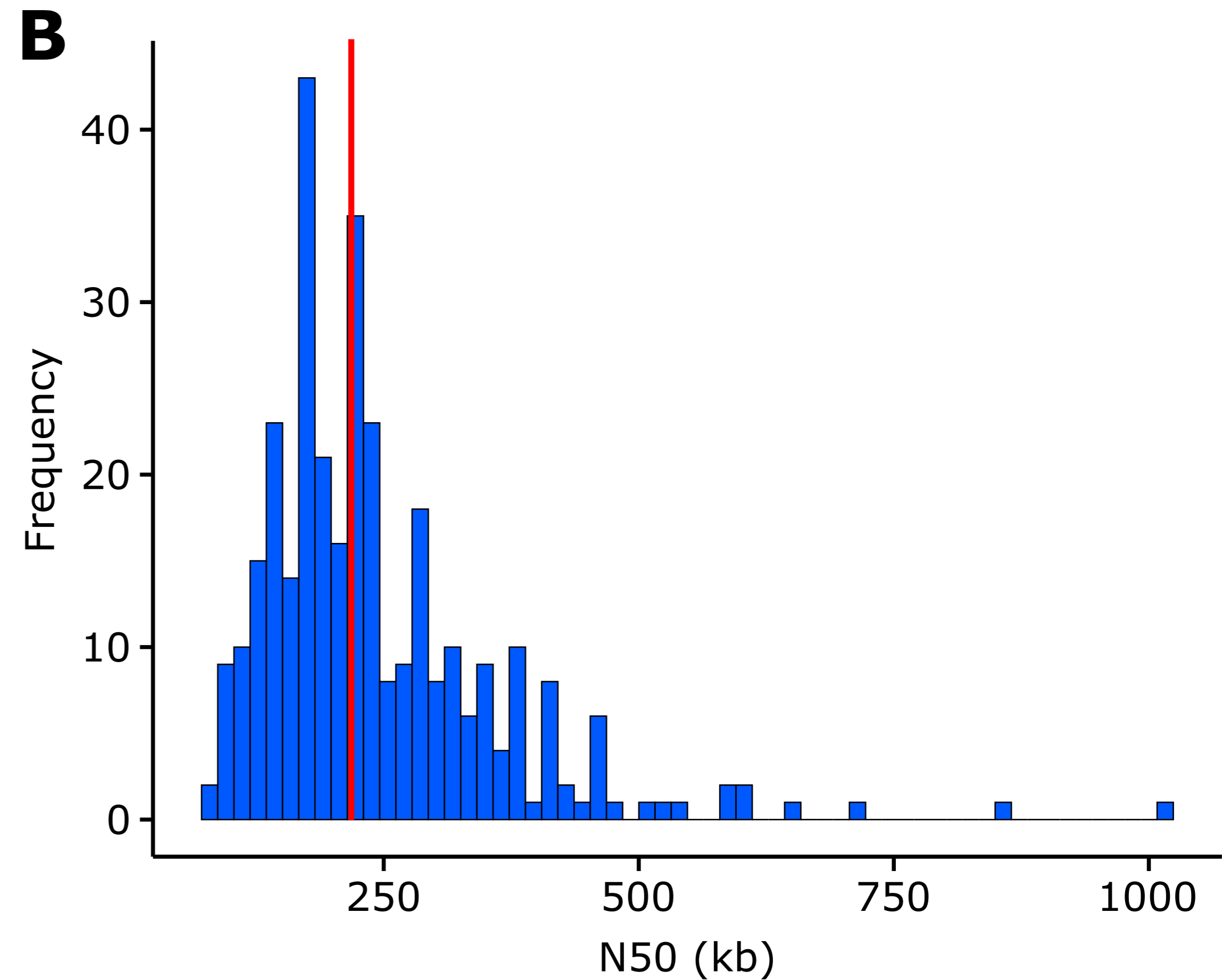

**Figure S1:** Assembly statistics of the *S. aureus* genomes

Supplement: Supplementary file 2 — Additional file 2: Fig. S1. Assembly statistics of the S. aureus genomes [file 12879_2021_6293_MOESM2_ESM.pdf]
