# Supplementary material for: Genomic epidemiology of methicillin-resistant and -susceptible Staphylococcus aureus from bloodstream infections
Source: BMC Infect Dis. 2021 Jun 21;21:589. doi: 10.1186/s12879-021-06293-3 (PMC8215799; doi:10.1186/s12879-021-06293-3)
Supplement: Supplementary file 4 — Additional file 4: Fig. S2. Pan-genome analysis of 323 S. aureus genomes. (a) Presence-absence matrix of gene clusters as determined by Roary, aligned to the phylogeny. Blue indicates presence. (b) Gene frequency histogram indicating the number of genomes each gene is present in. (c) The size of the pan-genome (blue), core genome (red), unique gene additions (purple) and new genes (green) as related to the number of individuals in the population. Proportional illustration of core, soft-core, shell, and cloud genes across the pangenome. [file 12879_2021_6293_MOESM4_ESM.pdf]

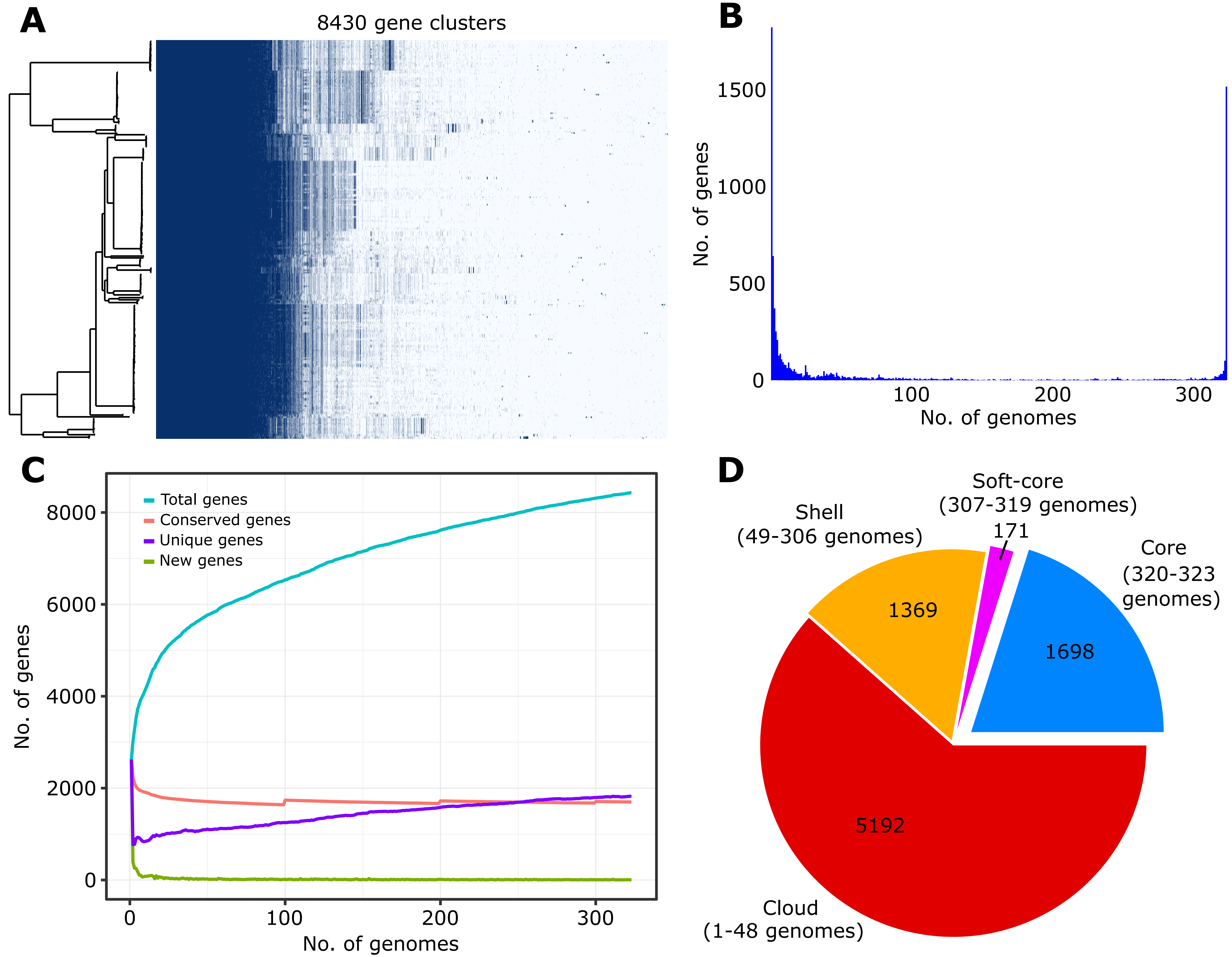

Figure S2: Pan-genome analysis of 323 *S. aureus* genomes. (a) Presence-absence matrix of gene clusters as determined by Roary, aligned to the phylogeny. Blue indicates presence. (b) Gene frequency histogram indicating the number of genomes each gene is present in. (c) The size of the pan-genome (blue), core genome (red), unique gene additions (purple) and new genes (green) as related to the number of individuals in the population. Proportional illustration of core, soft-core, shell, and cloud genes across the pangenome.
