# Supplementary material for: Genomic epidemiology of methicillin-resistant and -susceptible Staphylococcus aureus from bloodstream infections
Source: BMC Infect Dis. 2021 Jun 21;21:589. doi: 10.1186/s12879-021-06293-3 (PMC8215799; doi:10.1186/s12879-021-06293-3)
Supplement: Supplementary file 6 — Additional file 6: Fig. S3. Distribution of virulence genes using VirulenceFinder and the Virulence Factor Database (VFDB). Details are shown in Supplementary Table S4. [file 12879_2021_6293_MOESM6_ESM.pdf]

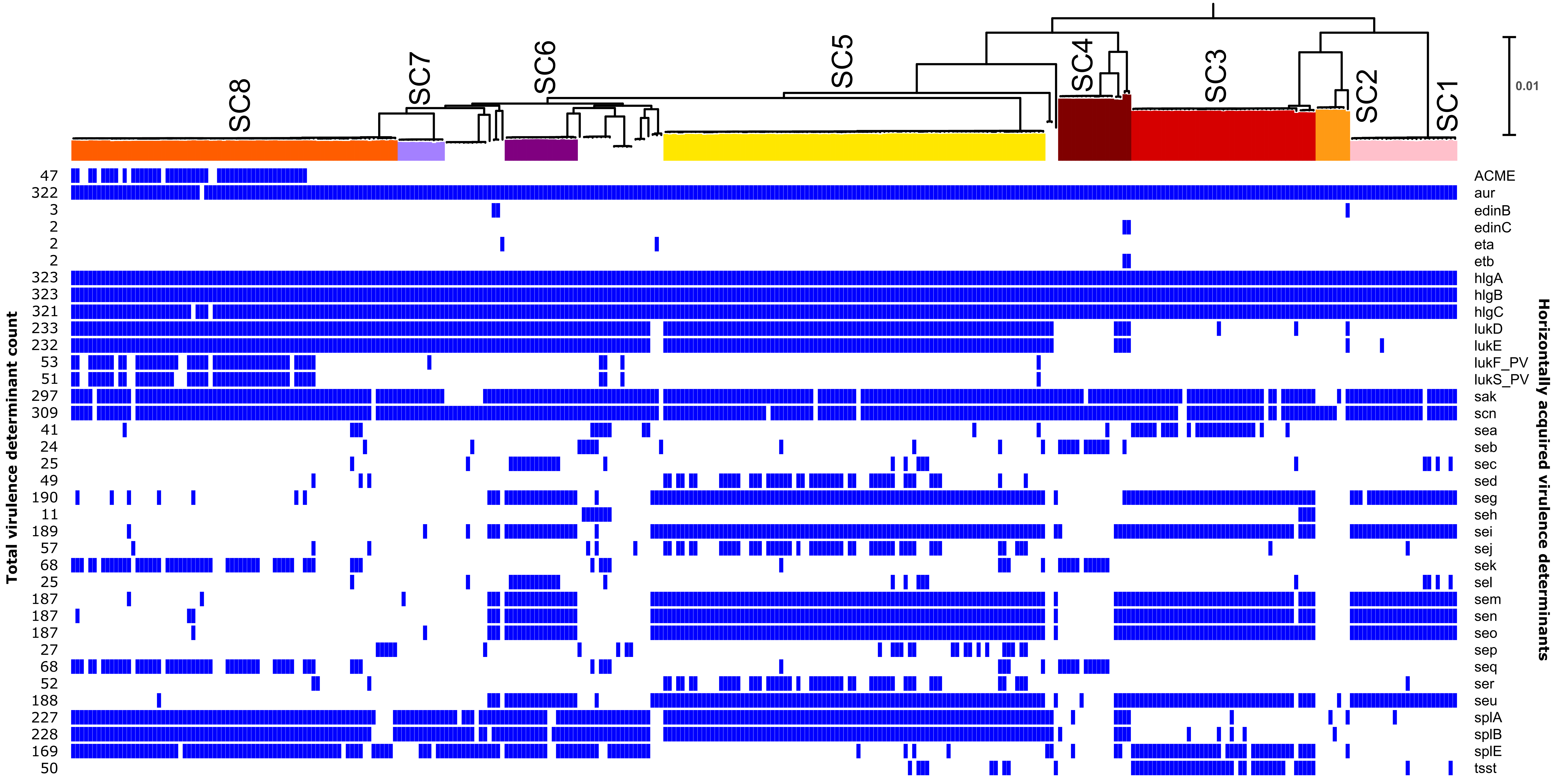

Figure S3: Distribution of virulence genes using VirulenceFinder and the Virulence Factor Database (VFDB). Details are shown in Supplementary Table S4.
