# Supplementary material for: Genomic epidemiology of methicillin-resistant and -susceptible Staphylococcus aureus from bloodstream infections
Source: BMC Infect Dis. 2021 Jun 21;21:589. doi: 10.1186/s12879-021-06293-3 (PMC8215799; doi:10.1186/s12879-021-06293-3)
Supplement: Supplementary file 8 — Additional file 8: Fig. S4. Bactdating statistical tests and MCMC trace plots. (a) Initial rooted phylogeny and correlation test between date and root-to-tip distance withing the phylogeny for CC5. (b) Bactdating trace plots constructed by periodic sampling over the MCMC runs for CC5. (c) Initial phylogeny and correlation test for CC8. (d) Bactdating trace plots for CC8. [file 12879_2021_6293_MOESM8_ESM.pdf]

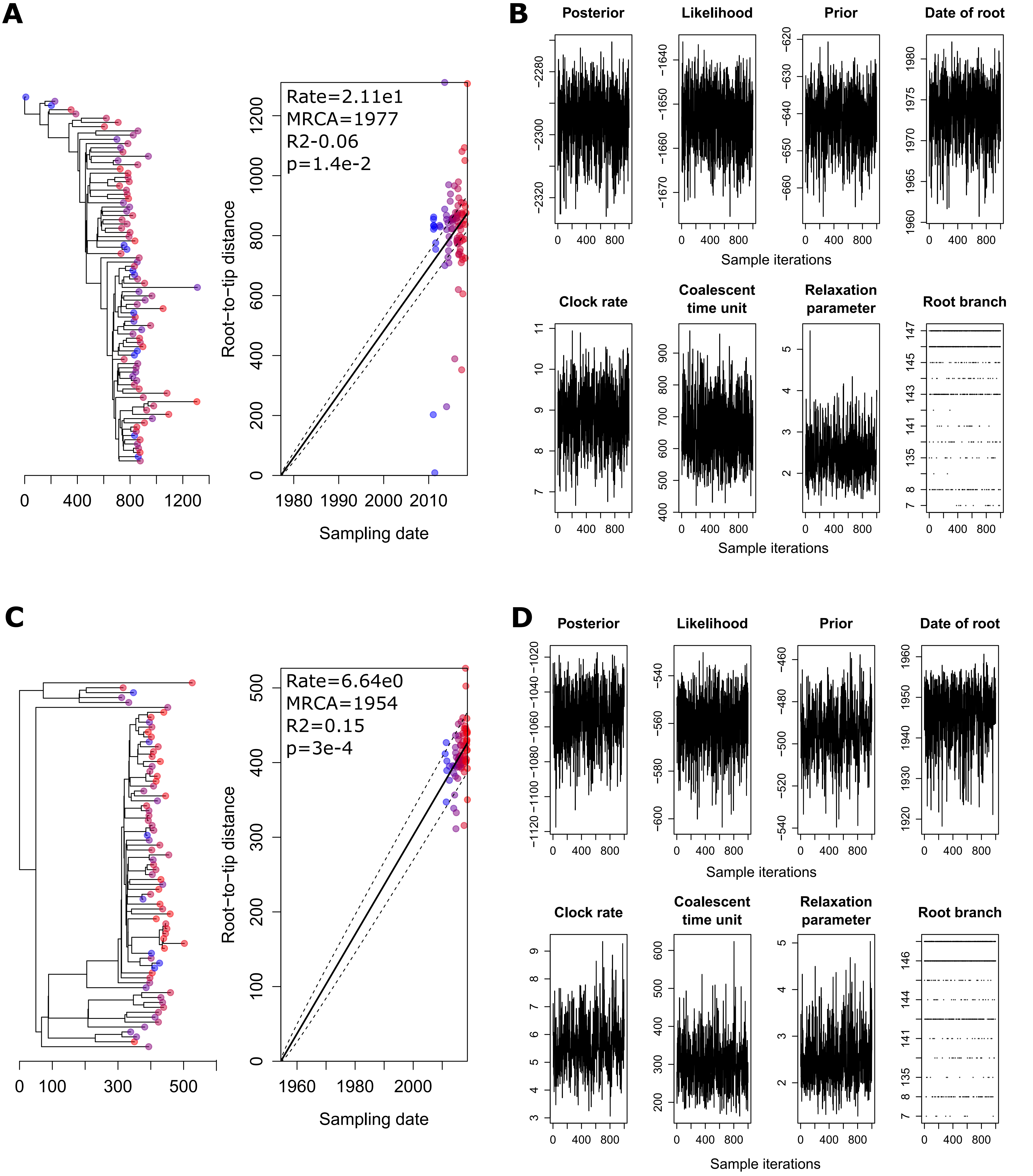

Figure S4: Bactdating statistical tests and MCMC trace plots. (a) Initial rooted phylogeny and correlation test between date and root-to-tip distance withing the phylogeny for CC5. (b) Bactdating trace plots constructed by periodic sampling over the MCMC runs for CC5. (c) Initial phylogeny and correlation test for CC8. (d) Bactdating trace plots for CC8.
